# Temperature-Responsive and Self-Healing Hydrogel: A Novel Approach to Combat Postoperative Adhesions

**DOI:** 10.3390/polym17141925

**Published:** 2025-07-12

**Authors:** Yujia Zhan, Xueshan Zhao, Changyuan He, Siwei Bi, Ruiqi Liu, Jun Gu, Bin Yan

**Affiliations:** 1 Department of Cardiovascular Surgery, West China Hospital, Sichuan University, Chengdu 610000, China; 2 National Engineering Laboratory for Clean Technology of Leather Manufacture, College of Biomass Science and Engineering, Sichuan University, Chengdu 610000, China; 3 Department of Burn and Plastic Surgery, West China Hospital, Sichuan University, Chengdu 610000, China

**Keywords:** postoperative adhesions, PEG-based hydrogel, biocompatibility, anti-adhesion

## Abstract

Postoperative adhesions are a prevalent complication following abdominal surgeries, often leading to significant clinical challenges. This study introduces an innovative solution utilizing a polyethylene glycol (PEG)-based triblock copolymer to form an injectable, self-healing hydrogel aimed at preventing these adhesions. The hydrogel, formulated with temperature-responsive and self-healing properties through the incorporation of poly (N-isopropyl acrylamide) (PNIPAM) and anion–pi interactions, was synthesized using reversible addition–fragmentation chain transfer (RAFT) polymerization. The hydrogel’s physical properties, biocompatibility, hemostatic effect, and anti-adhesive capabilities were rigorously tested through in vitro and in vivo experiments involving rat models. It demonstrated excellent biocompatibility, effective tissue adhesion, and robust hemostatic properties. Most notably, it exhibited significant anti-adhesive effects in a rat abdominal wall–cecum model, reducing adhesion formation effectively compared to controls. The PEG-based injectable hydrogel presents a promising approach for postoperative adhesion prevention. Its ability to gel in situ triggered by body heat, coupled with its self-healing properties, provides a substantial advantage in clinical settings, indicating its potential utility as a novel anti-adhesion material.

## 1. Introduction

Postoperative adhesion (PA) is a common complication in abdominal surgery, with an incidence rate exceeding 90%. It can lead to numerous conditions, such as abdominal pain, intestinal obstruction, infertility, and increased pelvic pain, thus elevating the complexity and complications of surgeries [1,2]. Therefore, the prevention and treatment of intra-abdominal adhesions hold significant clinical importance in the field of surgery and represent a critical issue.

The process of intra-abdominal adhesion formation is extremely complex. Following peritoneal injury, the body enters a stress state, with inflammatory exudation and the secretion of large amounts of fibrin on the wound surface, forming soluble polymers under the action of thrombin [3,4]. These polymers, due to a prolonged stay, come into full contact with blood coagulation factors, turning into insoluble polymers that bind with proteins to form a gel matrix composed of fibrin, situated between two damaged peritoneal surfaces [5]. In the early postoperative stage, the fibrin gel-like matrix is gradually replaced by granulation tissue containing fibroblasts, macrophages, and inflammatory cells [6]. Subsequently, as fibroblasts proliferate massively and mesothelial cells of the peritoneum transform into myofibroblasts, permanent adhesions are ultimately formed. Moreover, aside from foreign objects remaining from the surgical process, the main cause of adhesion formation is attributed to blood remnants within the body. In the normal peritoneal repair process, blood clots can be absorbed within 48 h [7]; however, after peritoneal damage, the exudation of large amounts of blood leads to the formation of fibrin from blood clots on the wound surface, promoting the occurrence of adhesions.

From the process of adhesion formation, it is known that trauma, inflammation, and infection can all cause adhesions [8]. Therefore, researchers have targeted each factor that contributes to the occurrence of adhesions, employing improvements in surgical methods, the use of drugs, and physical barrier materials to mitigate or prevent the formation of adhesions. However, adhesiolysis surgery inevitably causes new trauma, leading to more severe recurrent adhesions [9]. While some drugs can reduce the incidence of adhesions to some extent, others may not only fail to suppress adhesions but could also cause potential side effects [10,11]. Moreover, due to the presence of abdominal fluid, most drugs are rapidly cleared from the abdominal cavity, rendering them ineffective [10]. In recent years, physical barrier materials have become the most commonly used type of material [12,13,14]. They mainly serve as isolation materials to separate the abdominal wall from organ surfaces, preventing the deposition and adhesion of fibrous tissue. Commercial physical barrier materials (e.g., Seprafilm^®^) are acknowledged as current standards for adhesion prevention. Seprafilm is a representative solid membrane anti-adhesion material that has garnered widespread attention due to its biodegradable properties, eliminating the need for a second surgery [7]. However, this solid membrane has significant flaws. The complex shapes of human organs and the irregular geometric shapes of surgically created wounds mean that the solid membrane may not fully cover the wound tissue, affecting its anti-adhesion effectiveness [15]. Additionally, the stability of the solid membrane’s form makes it unsuitable for use in laparoscopic surgery for minimally invasive treatment [16]. Furthermore, solid films lack dynamic functions such as self-healing and stimulus responsiveness [17]. Designing an injectable self-healing hydrogel can effectively overcome these limitations.

In recent years, new hydrogels have achieved a functional combination of tissue adhesion and anti-postoperative adhesion properties, and have been continuously optimized to address the limitations of traditional commercial hydrogels [18,19]. Injectable gels, due to their unique combination of the free-flowing nature of liquid anti-adhesion materials and the physical barrier effect of solid membranes, have garnered widespread attention among researchers [20,21,22]. In particular, injectable hydrogels with self-healing properties can resist certain deformations, maintain their original shape and function, effectively extend their service life, and have significant advantages in effectively preventing postoperative adhesion. In addition, polyethylene glycol (PEG), known for its excellent biocompatibility, is commonly applied to wound surfaces to prevent adhesions [23,24]. PEG can inhibit the formation of initial adhesions and the development of existing fibrin bands. Polyethylene glycol diacrylate primarily prevents postoperative adhesions through three mechanisms: Firstly, high-viscosity PEG covers the damaged surface, preventing contact with adjacent organs. Secondly, PEG can aggregate with proteins within the peritoneum, delaying absorption and enhancing separation effects. Lastly, PEG induces the formation of peritoneal fluid, thereby reducing inflammatory responses and fibrinolysis, thus preventing the formation of postoperative adhesions [23,25]. Moreover, higher concentrations of PEG can increase tissue adhesion capability, which is crucial for adhesion prevention.

**Figure 1:**
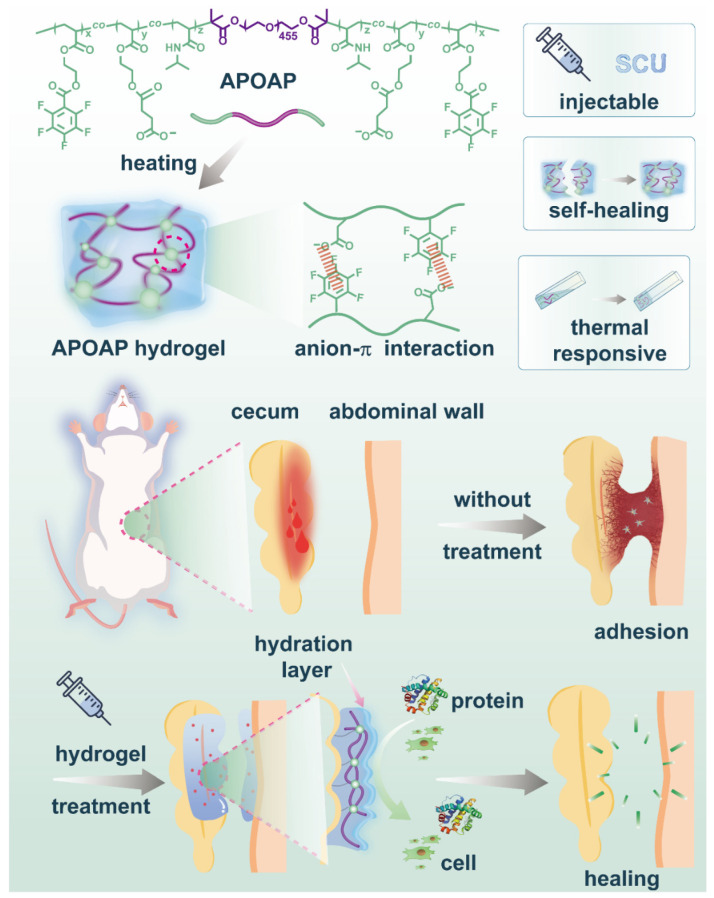
Biocompatibility, hemostatic activity, and anti-adhesion mechanism of APOAP.

Current clinical approaches for preventing postoperative adhesions primarily rely on surgical methods, drugs, and physical barrier materials. However, these methods have significant flaws. Moreover, existing injectable hydrogels lack dynamic self-healing capability, making them vulnerable to mechanical disruption during organ movement. To address these limitations, we prepared PEG-based tri-block polymer (poly {(N-isopropyl acryla-mide)-co-[4-(2-(acryloyloxy) ethoxy)-4-oxobutanoic acid]-co-(acryloyloxyethyl pen-tafluorobenzoate)}-b-PEO-b-Poly {(N-isopropyl acryla-mide)-co-[4-(2-(acryloyloxy)ethoxy)-4-oxobutanoic acid]-co-(acryloyloxyethyl pentafluoro-benzoate)}, APOAP) injectable self-healing hydrogels. This design aims to develop an injectable hydrogel that undergoes a rapid sol–gel transition at body temperature for seamless coverage of irregular lesions and autonomously repairs structural damage through reversible non-covalent bonds, thereby maintaining barrier integrity throughout the critical adhesion formation period. As illustrated in Figure 1, the synergistic combination of temperature-triggered gelling and dynamic self-healing mechanisms provides a dual-functional solution for persistent anti-adhesion protection.

## 2. Materials and Methods

### 2.1. Materials

Poly(ethylene glycol) (*M_n_* = 20,000, PEG, Sigma-Aldrich, Chengdu, China), and 2-hydroxyethyl acrylate (Huaxia Reagent, Chengdu, China) were used as received. 2-hydroxyethyl acrylate, pentafluorobenzoic acid, 4-Dimethylaminopyridine (DMAP), succinic anhydride, 1-(3-Dimethylaminopropyl)-3-ethylcarbodiimide hydrochloride (EDC·HCl), tetrahydrofuran (THF), dichloromethane (DCM), and other reagents were of analytical grade and purchased from Chengdu Kelong Chemical Reagent Co., Ltd. (Chengdu, China). 2,2′-Azobisisobutyronitrile (AIBN) and *N*-isopropylacrylamide (NIPAM) were purchased from Sigma-Aldrich and purified by recrystallization from methanol and benzene/n-hexane (65/35 *v*/*v*), respectively. The chain transfer agent (CTA), (S)-1-dodecyl-(S′)-(α,α′-dimethyl-α′′-acetic acid)-trithiocarbonate, and Macro-CTA agent RAFT-PEO_455_-RAFT were synthesized according to previous reports (Functional Polymers from Novel Carboxyl-Terminated Trithiocarbonates as Highly Efficient RAFT Agents; Thermoreversible Ion Gels with Tunable Melting Temperatures from Triblock and Pentablock Copolymers, Chengdu, China). Mouse dermal fibroblasts were purchased from American Type Culture Collection (ATCC, Manassas, VA, USA). Male Sprague Dawley (SD) rats (450 ± 50 g) were provided by Chengdu Dossy Experimental Animal Co., Ltd. (Chengdu, China). All of the animals were housed in specific-pathogen-free (SPF) conditions and were given ad libitum access to food and water. All animals would be in quarantine for a week before treatment. All animal procedures were performed following the protocol (Approval No. 20240104003) approved by the Institutional Animal Care and Treatment Committee of Sichuan University (Chengdu, China). All animals were treated humanely throughout the experimental period.

### 2.2. Synthesis of 4-(2-(Acryloyloxy)ethoxy)-4-oxobutanoic Acid (AEOA)

AEOA was synthesized following a reported procedure with some modifications [26]. Briefly, 2-hydroxyethyl acrylate (5.8 g 50 mmol) and DMAP (6.11 g, 50 mmol) were dissolved in 100 mL of DCM. Then, succinic anhydride (5.0 g, 50 mmol) was dissolved in 20 mL DCM and added to this solution dropwise in an ice bath with N_2_ bubbling. The resulting mixture was stirred at room temperature for 5 h. After that, the mixture was washed with 1 M of HCl solution (3 × 75 mL) and brine (1 × 75 mL). The organic phase was dried over anhydrous MgSO_4_, filtered to remove the desiccant, and concentrated under vacuum to obtain a light yellow oil product (yield: 39%). The 1H NMR (400 MHz, CDCl_3_) δ(ppm) showed the following results: 6.44 (dd, 1H, CH_2_=CH(C=O)O-), 6.14 (dd, 1H, CH_2_=CH(C=O)O-), 5.87 (dd, 1H, CH_2_=CH(C=O)O-), 4.40–4.29 (m, 4H, -OCH_2_CH_2_O-), 2.73–2.59 (m, 4H, -(C=O)CH_2_CH_2_(C=O)-).

### 2.3. Synthesis of Acryloyloxyethyl Pentafluorobenzoate (AOEPFB)

AOEPFB was created according to the reported procedure with some modifications [27]. Specifically, pentafluorobenzoic acid (6.7 g, 32 mmol), EDC·HCl (7.36 g, 38.4 mmol) and DMAP (0.04 g, 0.32 mmol) were dissolved in 100 mL of DCM. After that, 2-hydroxyethyl acrylate (4.09 g, 35.2 mmol) was added dropwise. The reaction was carried out at room temperature for 24 h. Then, the reaction solution was concentrated. The concentrated liquid was purified by column chromatography (petroleum ether/ethyl acetate 2:1) to obtain a light yellow liquid product (yield: 47%). The ^1^H NMR (400 MHz, CDCl_3_) δ (ppm) showed the following results: 6.45 (dd, 1H, CH_2_=CH(C=O)O-), 6.15 (dd, 1H, CH_2_=CH(C=O)O-), 5.89 (dd, 1H, CH_2_=CH(C=O)O-), 4.67–4.60 (m, 2H, CH_2_=CH(C=O)OCH_2_CH_2_O-), 4.52–4.45 (m, 2H, CH_2_=CH(C=O)OCH_2_CH_2_O-).

### 2.4. Synthesis of Poly {(N-Isopropyl acrylamide)-co-[4-(2-(acryloyloxy)ethoxy)-4-oxobutanoic acid]-co-(acryloyloxyethyl pentafluorobenzoate)}-b-PEO-b-Poly {(N-isopropyl acrylamide)-co-[4-(2-(acryloyloxy)ethoxy)-4-oxobutanoic acid]-co-(acryloyloxyethyl pentafluorobenzoate)} (APOAP)

APOAP was synthesized following the one-pot protocol in dioxane. Briefly, RAFT-PEO_455_-RAFT agent (0.518 g, 0.025 mmol), N-isopropylacrylamide (1.045 g, 9.25 mmol), AEOA (0.081 g, 0.375 mmol), AOEPFB (0.1163 g, 0.375 mmol), and azobisisobutyronitrile (0.002 g, 0.0125 mmol) were dissolved in 8 mL of dioxane. After purging N_2_ for 30 min, the whole system was stirred at 70 °C for 12 h. The polymerization was quenched by adding 5 mL of THF into the above mixture, and the resulting solution was added dropwise into a great amount of ethyl ether to precipitate the polymer out; the purification process was repeated twice. The polymer dried in vacuo. The composition of the resulting polymer was characterized by ^1^H NMR and was determined as Poly [NIPAM_175_-*co*-AEOA_8_-*co*-AOEPFB_7_]-b-PEO_455_-*b*-[NIPAM_175_-*co*-AEOA_8_-*co*-AOEPFB_7_], and the result is shown in the Supporting Information.

### 2.5. In Vitro Hemolysis Ratios of the APOAP Hydrogels

Hydrogel (100 μL) and an equal mass of gelatin sponge were soaked in 500 µL of rabbit blood containing sodium citrate (3.2%, blood/anticoagulant = 9/1) and placed on a shaker at 37 °C for 3 h. The above specimens were centrifuged for 10 min at 4000 rpm, and 200 µL of supernatant was collected and mixed with 5 mL of deionized water. UV–visible spectrophotometry was used to measure the absorbance at 540 nm. The hemolysis rate (%) = (OD_sample_ − OD_negative_)/(OD_positive_ − OD_negative_) × 100%, where OD represents the absorbance value at 540 nm. A total of 1% Triton X-100 was used as the positive control.

### 2.6. Blood Clotting Index of the APOAP Hydrogels

Hydrogel (100 μL) and an equal mass of gelatin sponge were placed in a 96-well plate. A total of 1 mL of CaCl_2_ (0.1 M) was added to 9 mL of citrated rabbit blood to activate the blood. A total of 50 µL of activated blood was dropped onto the surface of the hydrogel, and 5 mL of deionized water was used to dissolve the non-coagulated blood at different time points. The absorbance at 540 nm was measured using UV–visible spectrophotometry. The BCI was calculated using 50 µL of blood in 5 mL of deionized water as the reference: BCI (%) = ODsample/ODreference × 100%.

### 2.7. In Vitro Cytocompatibility

Hydrogel (100 μL) was placed in a 96-well plate, incubated for 4 h, and washed three times with PBS. Mouse dermal fibroblasts were seeded onto the surface of the hydrogel (100 µL per well, 6000 cells per well) and cultured in a 5% CO_2_, 37 °C incubator for 24 h and 48 h. Live/dead cell double staining experiments were conducted in a 6-well plate (300 µL per well, 50,000 cells per well). After treatment with the live/dead cell double staining reagent kit according to the instructions, cell growth behavior on the surface of the hydrogel was observed using an inverted fluorescence microscope (Olympus, BX51, Tokyo, Japan).

### 2.8. In Vivo Hemostasis Experiment in Rats

SD rats were selected for the experiment. A rat liver injury model was used to evaluate the hemostatic performance of different hemostatic materials. Rats were anesthetized with 2–3% isoflurane anesthesia, and anesthesia was maintained with a nasal mask during surgery. The abdomen was horizontally incised to expose the liver, and pre-weighed filter paper was placed under the organ. A 20 mm long piece of liver was cut from the bottom of the liver lobe to trigger bleeding. After ≈5 s without bleeding, hydrogel or gelatin sponge was applied to cover the bleeding site. The amount of bleeding was calculated by weighing the filter paper before and after blood absorption. Hemostasis time was also recorded.

### 2.9. In Vivo Anti-Adhesion Evaluation

Firstly, SD rats were anesthetized with 50 mg/kg pentobarbital sodium. The abdominal skin was shaved and disinfected with iodine. Then, a single incision of 4~5 cm was made along the midline of the abdomen using surgical scissors. The cecum was separated, and its serosal surface was gently rubbed with sterile surgical gauze until pinpoint bleeding occurred. A 1 cm × 2 cm peritoneal defect was created on the corresponding lateral side of the abdominal wall using a surgical blade. In the 15% hydrogel treatment group, 1 mL of 15% hydrogel was injected into the injured abdominal wall and cecum, without hemostasis. In the positive control group, 1 mL of commercial hydrogel was injected into the injured abdominal wall and cecum, without hemostasis. In the negative control group, the wound was sprayed with 1 mL of sterile saline. Each group consisted of 12 SD rats. After 7 and 14 days, 6 rats from each group were euthanized, the abdominal membranes were opened, and adhesion formation was observed. Photographs and scores of the adhesions formed between the cecum and abdominal wall were taken and recorded using Nair’s adhesion scoring system [28]. Subsequently, cecal and abdominal wall tissues from day 14 were collected and analyzed using H&E, Masson, and Van Gieson staining.

### 2.10. Statistical Analysis

All experimental data were expressed as mean ± standard deviation (SD). Tukey’s test and one-way ANOVA followed by Bonferroni’s multiple comparison tests were performed to analyze the data when appropriate. A value of *p* < 0.05 was set as statistically significant.

## 3. Results and Discussion

### 3.1. Preparation and Characterization of Hydrogel

The APOAP tri-block copolymer was synthesized via reversible addition fragmentation chain transfer (RAFT) polymerization (Figure S1) [29]. The synthesized copolymer was characterized by ^1^H NMR and gel permeation chromatography (GPC) (Mn = 60,300 g/mol, Mw = 77,200 g/mol, PDI = 1.28). As shown in Figure S2, the chemical shifts at 4.57, 4.20, 3.51, and 1.04 ppm are the characteristic peaks of pentafluorophenyl, carboxyl, PEO, and PNIPAM, respectively. The chemical shifts at 3.84 and 1.04 ppm are attributed to the last methyl hydrogen and methyl hydrogen of structural unit NIPAM; the chemical shift of methylene hydrogen near the polymer backbone in structural unit MAOES appeared at 4.20 ppm, and the characteristic absorption peaks of methylene hydrogen near the polymer backbone and near the pentafluorophenyl group in structural unit AOEPFB appeared at 4.57 and 4.57 ppm, respectively. The characteristic absorption peaks of methylidene hydrogen near the polymer backbone and near the pentafluorophenyl group in the structural unit AOEPFB appear at 4.57 and 4.20 ppm, respectively. The appearance of these peaks proves that the tri-block polymer containing NIPAM, MAOES, AOEPFB, and PEO in the main chain was successfully synthesized by RAFT polymerization. The ratio of the three structural units on the APOAP chain was calculated to be NIPAM:MAOES:AOEPFB = 349:15:13 by the integral area calculation of the ^1^H NMR spectrum. After characterization of the polymer, hydrogels were prepared by dissolving them in PBS solution at concentrations of 12.5 wt%, 15 wt%, and 17.5 wt%. Preliminary evaluation of their self-healing properties revealed that the Gel-12.5% hydrogel exhibited low modulus recovery efficiency and a decreasing trend in recovered modulus during cyclic strain step tests (300–1%), indicating poor self-healing performance. In contrast, both the Gel-15% and Gel-17.5% hydrogels demonstrated excellent self-healing properties (Figure S3). Based on the optimal self-healing performance and considerations of cost-effectiveness, the 15 wt% hydrogel (named Gel-15%) was selected for subsequent experiments.

**Figure 2:**
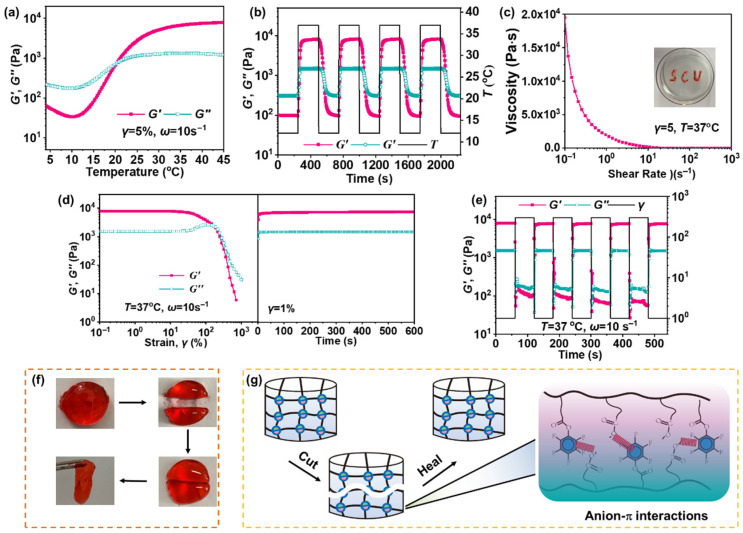
(**a**) Modulus variations with temperature (4 °C~45 °C) of hydrogel. (**b**) Modulus changes of hydrogels during multiple heating–cooling cycles. (**c**) Hydrogel viscosity at 37 °C with shear rates ranging from 1 to 1000 S^−1^. (**d**) Strain amplitude measurements of hydrogel at 37 °C (**left**) and time scan measurements at 1% strain demonstrate quick recovery from damage (**right**). (**e**) Cyclic strain step test demonstrates reproducible self-repair capabilities. (**f**) Optical images of hydrogel self-healing (stained with methyl orange). (**g**) Diagram demonstrating self-healing capability of hydrogel.

Temperature ramp experiments were performed on the Gel-15% hydrogel to determine its sol–gel transition temperature (*Ts-g*). As shown in Figure 2a, we can see that at a low temperature (4 °C), the storage modulus (*G*′) of Gel-15% is less than its loss modulus (*G*′′) and behaves as a sol–gel. As the temperature increases, the *G*′ of Gel-15% gradually becomes larger, exceeding the *G*′′ at 21 °C to reach the plateau region (basically an unchanged modulus) at around 33 °C. This indicates that at temperatures greater than 21 °C, Gel-14% behaves as a gel with a *Ts-g* of 21 °C. To demonstrate that this process is fully reversible, we performed dynamic temperature scanning measurements on Gel-15%. The results are shown in Figure 2b, where at 12 °C (<*Ts-g*), the *G*′ of Gel-15% is less than the *G*′′, and it behaves as a sol–gel. However, when the temperature was increased to 37 °C (>*Ts-g*), which is comparable to the human body temperature, the *G*′ of Gel-15% was greater than the *G*′′ and indicated a gel state. And this sol–gel state is completely reversible and reproducible with a change in temperature. These results indicate that hydrogels have excellent thermosensitive properties. In addition, the shear-thinning behavior of the hydrogel indicates that the hydrogel has fascinating injectable properties (Figure 2c).

As an implantable material, having self-healing properties can effectively avoid the trouble (e.g., infection) associated with hydrogel destruction and prolong the material’s lifetime [30]. Dynamic strain scans were performed on the Gel-15% hydrogel to measure the critical strain of the hydrogel. As shown in Figure 2d, the *G*′ of the hydrogel was equal to the *G*′′ at a strain of 160%, indicating that the critical strain of the hydrogel was 160%. Then, the hydrogel was subjected to step strain tests between a strain of 300% and 1%. As shown in Figure 2e, when the hydrogel was subjected to a strain (300%) greater than the critical strain, the *G*′ of the hydrogel was less than the *G*′′, indicating that the network of the hydrogel was destroyed. When subjected to a strain (1%) smaller than the critical strain, the *G*′ of the hydrogel was able to recover rapidly to the initial value, indicating that the mechanical properties of the hydrogel can recover autonomously. These results indicate that the hydrogels have good self-healing properties. The self-healing effect of Gel-15% was macroscopically demonstrated through a standardized incision–recovery test (Figure 2f). An intact hydrogel sample was cut in the middle with a paper cutter, and then the two pieces of hydrogel were brought into contact with each other at the incision to self-heal without applying any external force. Within 2 min, interfacial healing progressed sufficiently to allow the rejoined specimen to be lifted from one end while maintaining full structural integrity. The two pieces of hydrogel could be lifted up and the two pieces of hydrogel could be integrated together, and there were no problems with self-healing in the two pieces of hydrogel. The two hydrogels could be lifted and “integrated” together without breaking in the middle. As shown in Figure 2g, hydrogel promotes self-healing through anion–π interaction in hydrogels [31].

### 3.2. Adhesion Properties of Hydrogels

**Figure 3:**
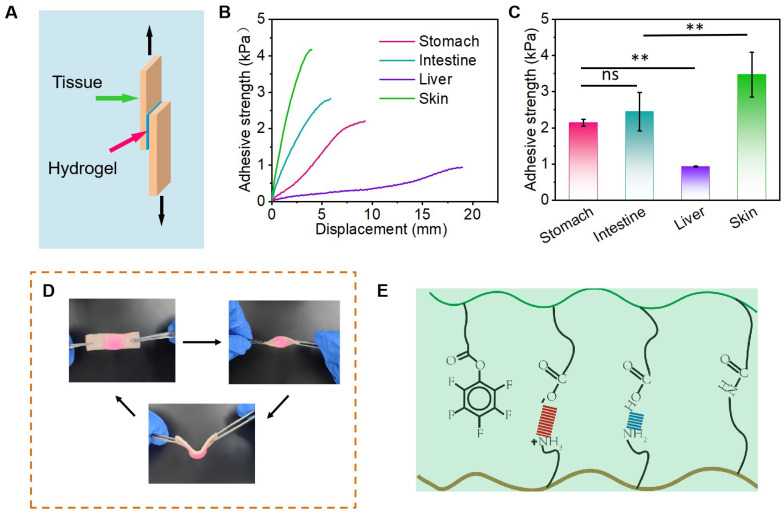
(**A**) Schematic of tissue adhesion test. (**B**) Stress–strain curve of APOAP hydrogel on different tissues. (**C**) Adhesion strength of APOAP hydrogel on different tissues. (**D**) Practical picture of adhesion of APOAP hydrogel on pig skin. (**E**) Schematic illustration of hydrogel adhesion mechanism. ** *p* < 0.01; ns, no significance.

In order to achieve in vivo hemostasis, hydrogels must possess clinical relevance to ensure their sustained presence at the lesion site [32]. Therefore, it is imperative for these hydrogels to exhibit adhesion capacity throughout the required duration of time [33]. The adhesion strength of Gel-15% on pig liver, pig stomach, pig intestine, and pig skin was tested using lap shear tensile tests (Figure 3A). As shown in Figure 3B, the adhesion strength of the hydrogels to the four organs in general showed that liver < stomach < intestine < skin, and in the case of Gel-15%, the adhesion strengths to the four organs mentioned above were 0.93 ± 0.01 kPa, 2.14 ± 0.10 kPa, 2.45 ± 0.53 kPa, and 3.47 ± 0.62 kPa, respectively. The Gel-15% adhered to the pig skin and maintained its initial shape after deformation such as twisting and bending without any peeling, which also proved that the hydrogel had better tissue adhesion (Figure 3C,D). The excellent adhesion properties of hydrogels can be primarily attributed to the formation of hydrogen bonds and electrostatic interactions between the carboxyl groups within the hydrogel and the skin (Figure 3E) [33].

### 3.3. Hemocompatibility, Cytocompatibility, and Hemostatic Properties of Hydrogels

**Figure 4:**
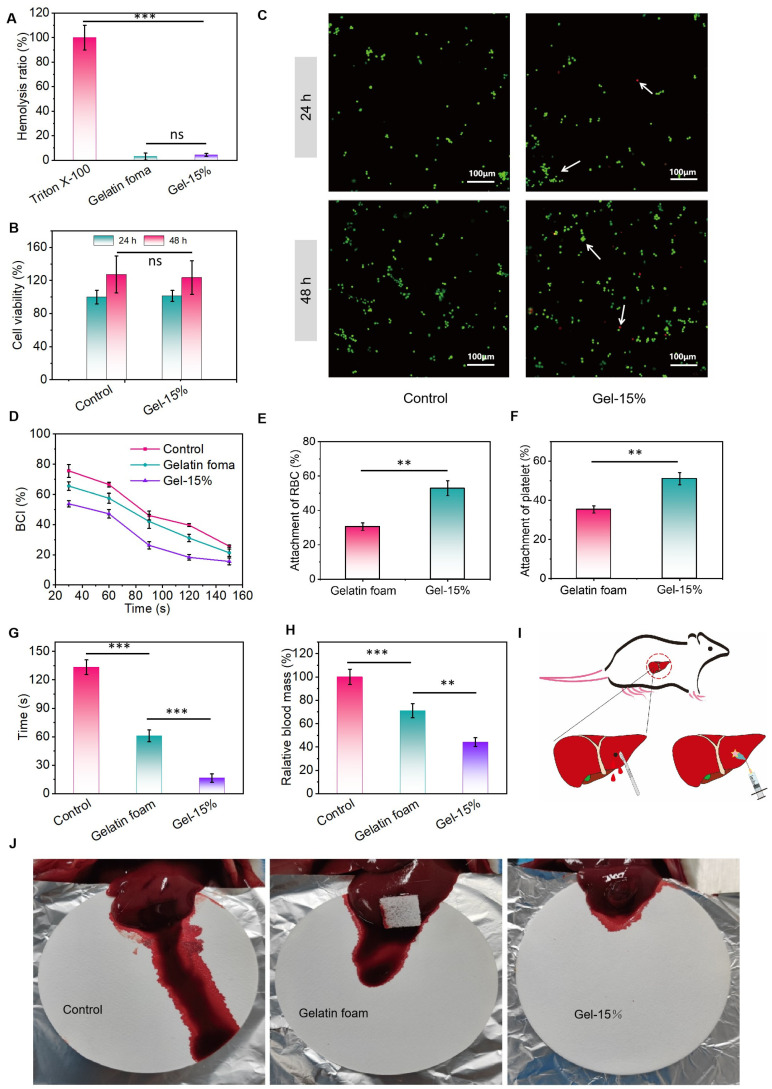
(**A**) Hemolysis ratio of hydrogel and gelatin foam. (**B**) Cell viability of L929 cells incubated with hydrogel. (**C**) Live/dead staining of L929 cells incubated with extracts from hydrogel for 24 and 48 h (White arrows: green cells: viable L929; red cells: apoptotic). (**D**) Blood clotting index (BCI) of hydrogel. (**E**) Erythrocyte adhesion rates. (**F**) Platelet adhesion rates. (**G**) Relative blood mass, and (**H**) hemostasis time of hydrogel and gelatin foam. (**I**) Scheme representation of mouse liver trauma model. (**J**) Bloodstain photographs of hydrogel and gelatin foam. ** *p* < 0.01, *** *p* < 0.001; ns, no significance.

Since hydrogels are to be in direct contact with blood, in vitro hemolysis experiments were performed to determine the hemocompatibility of hydrogels. The hemolysis rate represents the degree of destruction of red blood cells, with larger values indicating more severe destruction of red blood cells. Tritone X-100 and PBS were set as the positive and negative control groups, respectively. As shown in Figure 4A, the hemolysis rates were 3.00 ± 2.81% and 4.26 ± 1.24% for the gelatin foam group and Gel-15%, respectively, and were kept at a low level, which indicates that the hydrogel group had good hemocompatibility. In addition, the cytocompatibility of the hydrogel was determined using the CCK-8 assay. The cellular activity of the hydrogel was maintained at 98.31% ± 6.54% (24 h) and 121.73% ± 18.59% (48 h) after co-culturing with L929 cells for 24 and 48 h, respectively, indicating that the hydrogel has low cytotoxicity (Figure 4B). The same results were observed in the live/dead staining assay of the cells (Figure 4C).

In order to investigate the coagulation properties of hydrogels in vitro, whole blood coagulation experiments were performed to determine the value of the blood clotting index (BCI), with a low BCI indicating a fast coagulation rate (ultrafast in situ-forming halloysite nanotube-doped chitosan/oxidized dextran hydrogels for hemostasis and wound repair). As shown in Figure 4D, the BCI values of the blank, gelatin sponge, and hydrogel groups all decreased over time, indicating that the rabbit blood was clotting. At different time points, the BCI values of the hydrogel group were lower than those of the gelatin foam and blank groups, indicating that the hydrogel was effective at promoting blood coagulation. Since erythrocyte and platelet aggregation play a dominant role in initial hemostasis (sandwich-like fibers/sponge composite combining chemotherapy and hemostasis for efficient postoperative prevention of tumor recurrence and metastasis), in order to investigate the reason why the hydrogel group had a lower BCI, erythrocyte adhesion and platelet adhesion experiments were performed. The erythrocyte adhesion rates of the gelatin foam group and Gel-15% were 30.52 ± 2.17% and 52.91 ± 4.38%, respectively (Figure 4E), and the adhesion rates of the above two groups to platelets were 35.27 ± 1.85% and 51.05 ± 3.11% (Figure 4F). These results indicate that the hydrogel group exhibited favorable adhesion to erythrocytes and platelets, which is useful for hemostasis.

Based on the excellent in vitro coagulation ability of Gel-15% hydrogels, we hypothesized that the hydrogels were effective at hemostasis in vivo. Using a rat liver hemorrhage model, the relative blood loss as well as the hemostasis time when the hydrogel was applied to the bleeding site were evaluated. Compared to the bleeding in the blank group (99.94 ± 7.53%) and gelatin foam group (70.05 ± 6.62%), the relative blood loss of Gel-15% (44.38 ± 4.23%) was significantly reduced (Figure 4G), and the hydrogels also showed a reduction in hemostatic time to 59.25 ± 4.57 s, 30.50 ± 4.43 s, and 17.50 ± 4.20 s, respectively (Figure 4H). In addition, the gelatin foam and Gel-15% hydrogel were both effective at stopping bleeding and bonding tightly to the liver after injection into the wound site, and no secondary bleeding was observed (Figure 4I,J). These results clearly demonstrate the excellent potential of the Gel-15% hydrogel for in situ hemostasis in vivo.

### 3.4. In Vivo Anti-Adhesive Efficacy of APOAP Hydrogel

**Figure 5:**
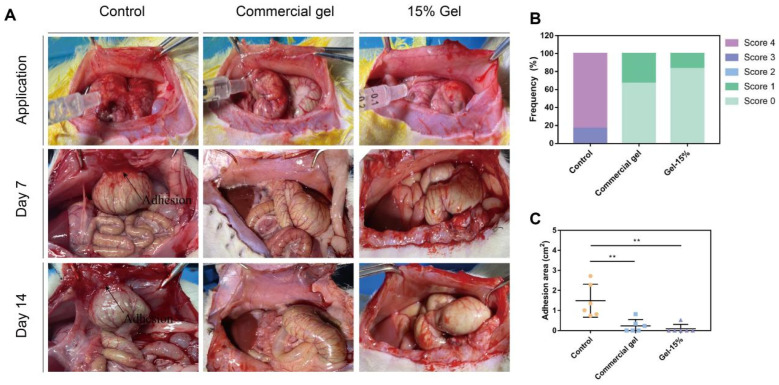
Prevention of postoperative abdominal adhesions in a rat abdominal wall–cecum adhesion model. (**A**) The rat abdominal wall–cecum adhesion model and the hydrogel application. Adhesions were observed in the control group (rats treated with saline only) on days 7 and 14. No adhesion was detected between the defected wall and abraded cecum in rats treated with hydrogel 15% gel on days 7 and 14. (**B**) Nair’s adhesion scores (0–4) in different groups of rats: control, commercial gel, and 15% gel after 14 days of surgery. (Score 2 was not observed in any group.) (**C**) The adhesion area in different groups of rats: control, commercial gel, and 15% gel after 14 days of surgery. ** *p* < 0.01.

We have successfully prepared hydrogels of different ratios. Through characterization and biocompatibility testing, we found that the 15% AP hydrogel possesses a uniform pore size distribution, convenient gelation time, good swelling performance, and excellent hemostatic ability. Subsequently, we constructed a SD rat abdominal wall–cecum adhesion model [34], primarily involving the creation of localized bleeding points on the cecum and scraping a similar-sized area of damage on the opposite abdominal wall (Figure 5A).

Post-surgical abdominal adhesions form within 3 to 14 days [35,36]. Currently, it is crucial to intervene with anti-adhesion measures within the biodegradation period (3–14 days) in clinical settings [35]. In this critical period for adhesion, post-operative abdominal adhesion barriers are designed to form a barrier to prevent excessive tissue formation at the injury site [37]. Therefore, after the surgical procedure, we opened the animals’ abdomens on post-operative days 7 and 14 to observe adhesions, recording the situation using Nair’s adhesion scoring system [28]. The results showed that on day 14, the control group exhibited varying degrees of severe adhesions, while the commercial gel group and Gel-15% group effectively prevented the formation of adhesions, significantly reducing the degree of adhesion grading (Figure 5B) and the area of adhesion (Figure 5C). In the control group, 75% scored a 4, while in the commercial gel and Gel-15% groups, the proportion of grade 4 scores was 37.5% and 12.5%, respectively, showing a clear anti-adhesion effect. These results indicate that our hydrogel itself has a good anti-PA capability and can play a role during the critical period of PA formation.

**Figure 6:**
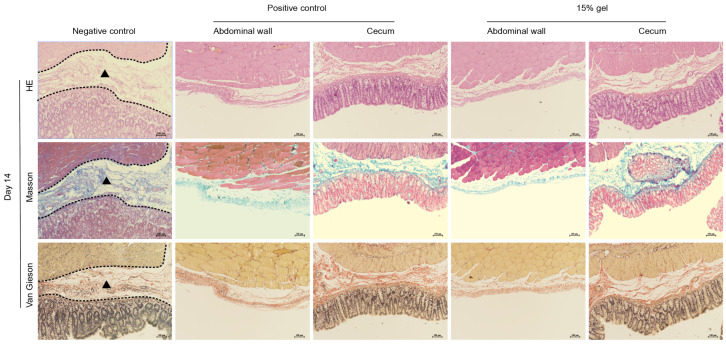
Histological evaluation of cecal wall and peritoneum for control, commercial gel, and Gel-15% hydrogel. HE, Masson’s trichrome, and Van Gieson stained showed severe adhesion in control group after 2 weeks of treatment. No adhesion was found in rats treated with Gel-15% hydrogel and commercial gel. Conditions of cecum and abdominal wall were similar to those of normal healthy rats. Collagen deposited in negative control sample ensured strong adhesion between cecum and cellular wall of abdomen. Triangle: adhesion area.

During the process of adhesion repair, histological characteristics such as decreased infiltration of inflammatory cells, disappearance of adhesion bands, repair of mesothelial cells, and reduced collagen deposition are observed [38]. Therefore, we analyzed the anti-adhesion effect of hydrogels from a histological perspective. On day 14, wound tissue samples were collected and observed histologically using hematoxylin and eosin (H&E) staining, Masson’s trichrome staining, and Van Gieson staining. In comparison, tissue sections from the negative control group showed adhesions of connective tissue to varying degrees at the injured cecum muscle tissue and peritoneum, with Masson’s trichrome staining revealing increased collagen deposition in the adhesion bands, dense collagen fibers, giant multinucleated cells, and a few scattered neutrophils near the adhesion bands indicated by the dashed line area, while the 15% gel group’s tissue sections showed reduced collagen deposition, no visible adhesion areas, and complete reconstruction of the epithelial structure of the abdominal wall and cecum (Figure 6). These results indicate that Gel-15% hydrogel can remain in situ and exhibit anti-adhesive properties during the critical period of PA formation.

## 4. Conclusions

In conclusion, we have successfully prepared an injectable self-healing hydrogel with a PEG-based triblock polymer for postoperative prevention of tissue adhesion. The PNIPAM segments incorporated into the polymer imparted excellent temperature-responsive properties to the hydrogel, enabling in situ gelation triggered by body heat, a facile application, and effective coverage of irregular damaged areas. At the same time, the anion–pi interactions in the hydrogel can promote the self-healing of the hydrogel, effectively extending the service life of the material. It is noted that the prepared hydrogels have excellent biocompatibility and good adhesion to tissues. In addition, the prepared hydrogel has a strong hemostatic effect and can effectively manage postoperative bleeding. More importantly, the prepared hydrogel showed excellent anti-adhesion effects in the rat abdominal wall–cecum adhesion model. Therefore, the prepared APOAP hydrogel has great potential for clinical application in postoperative anti-adhesion.

## Data Availability

The original contributions presented in this study are included in the article/Supplementary Material. Further inquiries can be directed to the corresponding authors.

## Supplementary Materials

The following supporting information can be downloaded at: https://www.mdpi.com/article/10.3390/polym17141925/s1, Figure S1: Synthesis route of the ABA triblock copolymer APOAP; Figure S2: Characteristic peaks of the ABA triblock copolymer APOAP; Figure S3: Comparison of self-healing properties of hydrogels with different concentrations.

## Abbreviations

The following abbreviations are used in this manuscript:

PNIPAM	Poly (N-isopropyl acrylamide)
PEG	Polyethylene glycol
RAFT	Reversible addition–fragmentation chain transfer
PA	Postoperative adhesion
APOAP	Poly {(N-isopropyl acrylamide)-co-[4-(2-(acryloyloxy) ethoxy)-4-oxobutanoic acid]-co-(acryloyloxyethyl pentafluorobenzoate)}-b-PEO-b-Poly {(N-isopropyl acrylamide)-co-[4-(2-(acryloyloxy)ethoxy)-4-oxobutanoic acid]-co-(acryloyloxyethyl pentafluorobenzoate)}
DMAP	4-Dimethylaminopyridine
EDC·HCl	1-(3-Dimethylaminopropyl)-3-ethylcarbodiimide hydrochloride
THF	Tetrahydrofuran
DCM	Dichloromethane
AIBN	2,2′-Azobisisobutyronitrile
NIPAM	N-isopropylacrylamide
CTA	Chain transfer agent
ATCC	American type culture collection
SPF	Specific-pathogen-free
SD	Standard deviation
GPC	Gel permeation chromatography
H&E	Hematoxylin and eosin

## References

[1] Wang A.Y., Lam C.W., Wang M., Woo J., Chan I.H., Lui S.F., Sanderson J.E., Li P.K. (2005). Circulating soluble vascular cell adhesion molecule 1: Relationships with residual renal function, cardiac hypertrophy, and outcome of peritoneal dialysis patients. Am. J. Kidney Dis. Off. J. Natl. Kidney Found..

[2] Moris D., Chakedis J., Rahnemai-Azar A.A., Wilson A., Hennessy M.M., Athanasiou A., Beal E.W., Argyrou C., Felekouras E., Pawlik T.M. (2017). Postoperative Abdominal Adhesions: Clinical Significance and Advances in Prevention and Management. J. Gastrointest. Surg..

[3] Soltany S. (2021). Postoperative peritoneal adhesion: An update on physiopathology and novel traditional herbal and modern medical therapeutics. Naunyn-Schmiedeberg’s Arch. Pharmacol..

[4] Duron J.J. (2007). Postoperative intraperitoneal adhesion pathophysiology. Color. Dis. Off. J. Assoc. Coloproctology Great Br. Irel..

[5] Capella-Monsonís H., Kearns S., Kelly J., Zeugolis D.I. (2019). Battling adhesions: From understanding to prevention. BMC Biomed. Eng..

[6] Ensan B., Bathaei P., Nassiri M., Khazaei M., Hassanian S.M., Abdollahi A., Ghorbani H.R., Aliakbarian M., Ferns G.A., Avan A. (2022). The Therapeutic Potential of Targeting Key Signaling Pathways as a Novel Approach to Ameliorating Post-Surgical Adhesions. Curr. Pharm. Des..

[7] Chen J., Tang X., Wang Z., Perez A., Yao B., Huang K., Zhang Y., King M.W. (2023). Techniques for navigating postsurgical adhesions: Insights into mechanisms and future directions. Bioeng. Transl. Med..

[8] Hellebrekers B.W., Kooistra T. (2011). Pathogenesis of postoperative adhesion formation. Br. J. Surg..

[9] ten Broek R.P., Issa Y., van Santbrink E.J., Bouvy N.D., Kruitwagen R.F., Jeekel J., Bakkum E.A., Rovers M.M., van Goor H. (2013). Burden of adhesions in abdominal and pelvic surgery: Systematic review and met-analysis. BMJ (Clin. Res. Ed.).

[10] Kucukozkan T., Ersoy B., Uygur D., Gundogdu C. (2004). Prevention of adhesions by sodium chromoglycate, dexamethasone, saline and aprotinin after pelvic surgery. ANZ J. Surg..

[11] Yang L., Li Z., Chen Y., Chen F., Sun H., Zhao M., Chen Y., Wang Y., Li W., Zeng L. (2022). Elucidating the Novel Mechanism of Ligustrazine in Preventing Postoperative Peritoneal Adhesion Formation. Oxidative Med. Cell. Longev..

[12] Fang Y., Huang S., Gong X., King J.A., Wang Y., Zhang J., Yang X., Wang Q., Zhang Y., Zhai G. (2023). Salt sensitive purely zwitterionic physical hydrogel for prevention of postoperative tissue adhesion. Acta Biomater..

[13] Li J., Yu H., Kang Y., Niu K., Wang M., Jiang Y., Jiang N., Ding Z., Gan Z., Yu Q. (2024). STING Membrane Prevents Post-Surgery Tissue Adhesion and Tumor Recurrence of Colorectal Cancer. Adv. Mater..

[14] Li J.C., Wu Z., Jiao Z.X., Wang Y., Wang Z.L., Guo M., Li G., Wang L.Q., Zhang P.B. (2024). A rapid crosslinking injectable polygalacturonic acid barrier modified with zwitterion bottlebrush for preventing postoperative adhesion. Chem. Eng. J..

[15] Brochhausen C., Schmitt V.H., Planck C.N., Rajab T.K., Hollemann D., Tapprich C., Krämer B., Wallwiener C., Hierlemann H., Zehbe R. (2012). Current strategies and future perspectives for intraperitoneal adhesion prevention. J. Gastrointest. Surg. Off. J. Soc. Surg. Aliment. Tract..

[16] Ouaïssi M., Gaujoux S., Veyrie N., Denève E., Brigand C., Castel B., Duron J.J., Rault A., Slim K., Nocca D. (2012). Post-operative adhesions after digestive surgery: Their incidence and prevention: Review of the literature. J. Visc. Surg..

[17] Liu B., Kong Y., Alimi O.A., Kuss M.A., Tu H., Hu W., Rafay A., Vikas K., Shi W., Lerner M. (2023). Multifunctional Microgel-Based Cream Hydrogels for Postoperative Abdominal Adhesion Prevention. ACS Nano.

[18] Ma P., Liang W., Huang R., Zheng B., Feng K., He W., Huang Z., Shen H., Wang H., Wu D. (2024). Super-Structured Wet-Adhesive Hydrogel with Ultralow Swelling, Ultrahigh Burst Pressure Tolerance, and Anti-Postoperative Adhesion Properties for Tissue Adhesion. Adv. Mater..

[19] Peng W., Lai Y., Jiang Y., Zhang Y., Kan Z., Dai C., Shen J., Liu P. (2025). Charge balance transition enabled Janus hydrogel for robust wet-tissue adhesion and anti-postoperative adhesion. Bioact. Mater..

[20] Gao J., Wen J., Hu D., Liu K., Zhang Y., Zhao X., Wang K. (2022). Bottlebrush inspired injectable hydrogel for rapid prevention of postoperative and recurrent adhesion. Bioact. Mater..

[21] Liang Y., Xu H., Li Z., Zhangji A., Guo B. (2022). Bioinspired Injectable Self-Healing Hydrogel Sealant with Fault-Tolerant and Repeated Thermo-Responsive Adhesion for Sutureless Post-Wound-Closure and Wound Healing. Nanomicro. Lett..

[22] Yang Y., He G., Pan Z., Zhang K., Xian Y., Zhu Z., Hong Y., Zhang C., Wu D. (2024). An Injectable Hydrogel with Ultrahigh Burst Pressure and Innate Antibacterial Activity for Emergency Hemostasis and Wound Repair. Adv. Mater..

[23] Hemati H., Haghiralsadat F., Hemati M., Sargazi G., Razi N. (2023). Design and Evaluation of Liposomal Sulforaphane-Loaded Polyvinyl Alcohol/Polyethylene Glycol (PVA/PEG) Hydrogels as a Novel Drug Delivery System for Wound Healing. Gels.

[24] Shen Y., Xu G., Huang H., Wang K., Wang H., Lang M., Gao H., Zhao S. (2021). Sequential Release of Small Extracellular Vesicles from Bilayered Thiolated Alginate/Polyethylene Glycol Diacrylate Hydrogels for Scarless Wound Healing. ACS Nano.

[25] Li J., Feng X., Liu B., Yu Y., Sun L., Liu T., Wang Y., Ding J., Chen X. (2017). Polymer materials for prevention of postoperative adhesion. Acta Biomater..

[26] Olivier S., Derue L., Geffroy B., Maindron T., Ishow E. (2015). Small molecule-based photocrosslinkable fluorescent materials toward multilayered and high-resolution emissive patterning. J. Mater. Chem. C.

[27] Morgese G., Siegmann K., Winkler M. (2024). Specific, nondestructive, and durable adhesion primer for polyolefins. J. Coat. Technol. Res..

[28] Nair S.K., Bhat I.K., Aurora A.L. (1974). Role of proteolytic enzyme in the prevention of postoperative intraperitoneal adhesions. Arch. Surg..

[29] Keddie D.J. (2014). A guide to the synthesis of block copolymers using reversible-addition fragmentation chain transfer (RAFT) polymerization. Chem. Soc. Rev..

[30] Zhang X., Liang Y., Huang S., Guo B. (2024). Chitosan-based self-healing hydrogel dressing for wound healing. Adv. Colloid Interface Sci..

[31] Li Z., Lu J., Ji T., Xue Y., Zhao L., Zhao K., Jia B., Wang B., Wang J., Zhang S. (2024). Self-Healing Hydrogel Bioelectronics. Adv. Mater..

[32] Montazerian H., Davoodi E., Baidya A., Baghdasarian S., Sarikhani E., Meyer C.E., Haghniaz R., Badv M., Annabi N., Khademhosseini A. (2022). Engineered Hemostatic Biomaterials for Sealing Wounds. Chem. Rev..

[33] Ouyang C., Yu H., Wang L., Ni Z., Liu X., Shen D., Yang J., Shi K., Wang H. (2023). Tough adhesion enhancing strategies for injectable hydrogel adhesives in biomedical applications. Adv. Colloid Interface Sci..

[34] Poehnert D., Abbas M., Kreipe H.H., Klempnauer J., Winny M. (2015). High reproducibility of adhesion formation in rat with meso-stitch approximation of injured cecum and abdominal wall. Int. J. Med. Sci..

[35] Sultana T., Van Hai H., Park M., Lee S.Y., Lee B.T. (2020). Controlled release of Mitomycin C from modified cellulose based thermo-gel prevents post-operative de novo peritoneal adhesion. Carbohydr. Polym..

[36] Lee J.W., Park J.Y., Park S.H., Kim M.J., Song B.R., Yun H.W., Kang T.W., Choi H.S., Kim Y.J., Min B.H. (2018). Cross-linked electrospun cartilage acellular matrix/poly(caprolactone-co-lactide-co-glycolide) nanofiber as an antiadhesive barrier. Acta Biomater..

[37] Hindocha A., Beere L., Dias S., Watson A., Ahmad G. (2015). Adhesion prevention agents for gynaecological surgery: An overview of Cochrane reviews. Cochrane Database Syst. Rev..

[38] Hu Q., Xia X., Kang X., Song P., Liu Z., Wang M., Lu X., Guan W., Liu S. (2021). A review of physiological and cellular mechanisms underlying fibrotic postoperative adhesion. Int. J. Biol. Sci..

