# Supplementary material for: Temperature-Responsive and Self-Healing Hydrogel: A Novel Approach to Combat Postoperative Adhesions
Source: Polymers (Basel). 2025 Jul 12;17(14):1925. doi: 10.3390/polym17141925 (PMC12300532; doi:10.3390/polym17141925)
Supplement: Supplementary file 1 [file polymers-17-01925-s001.zip › polymers-3703093-supplementary.pdf]

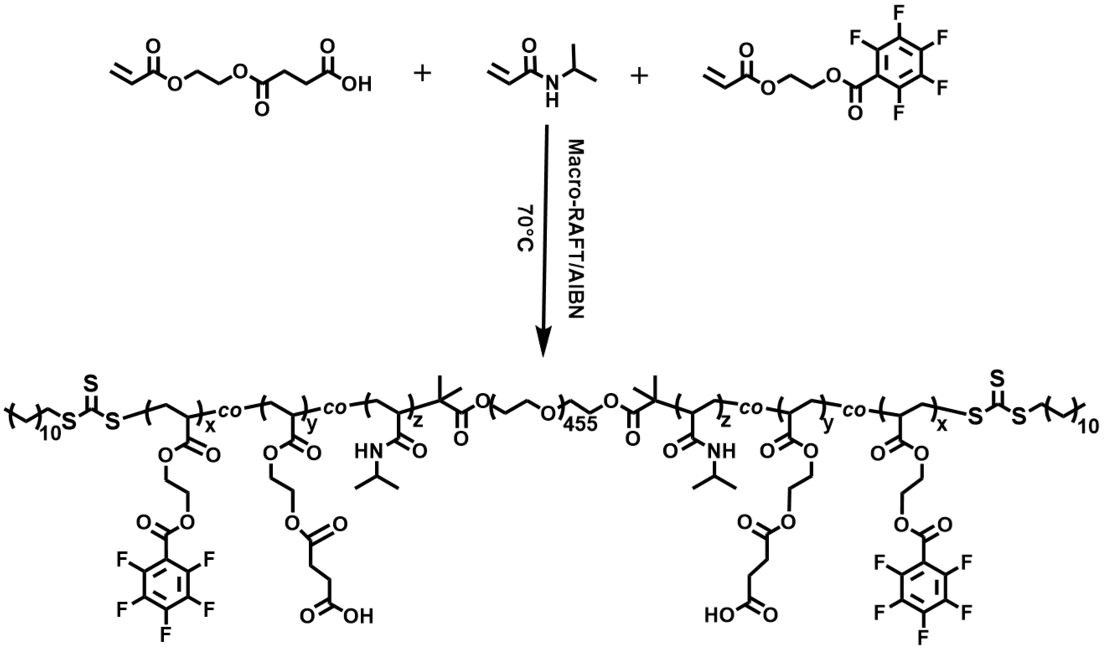

Figure S1. Synthesis route of the ABA triblock copolymer APOAP.

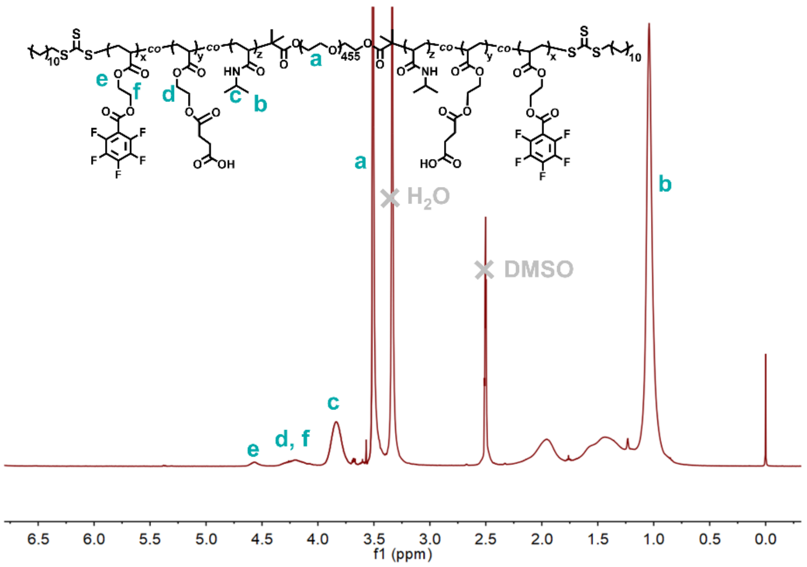

Figure S2. NMR spectra of APOAP tri-block copolymer.

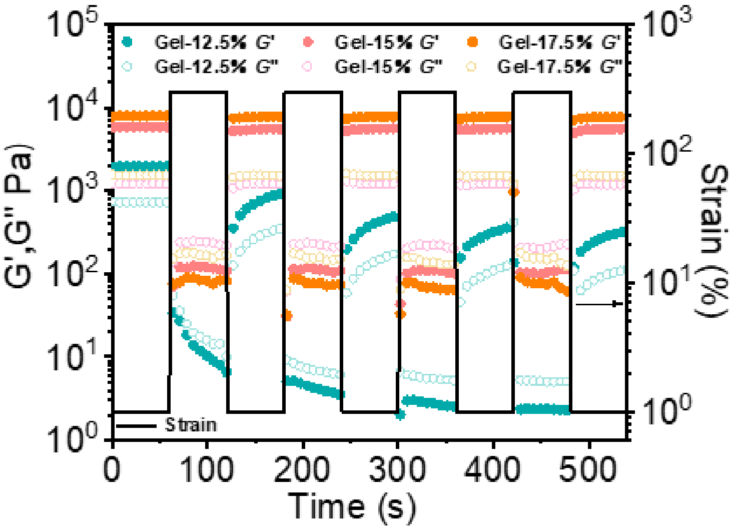

Figure S3. Comparison of self-healing properties of hydrogels with different concentrations
